# Who is actually asked about their mental health in pregnancy and the postnatal period? Findings from a national survey

**DOI:** 10.1186/s12888-016-1029-9

**Published:** 2016-09-15

**Authors:** Maggie Redshaw, Jane Henderson

**Affiliations:** Nuffield Department of Population Health, Policy Research Unit in Maternal Health and Care, National Perinatal Epidemiology Unit, University of Oxford, Old Road Campus, Headington, Oxford, OX3 7LF UK

**Keywords:** Mental health, Pregnancy, Postnatal, Maternal wellbeing

## Abstract

**Background:**

Pregnancy and the postnatal period is a period of potential vulnerability for women and families. It is UK policy that all women are asked about their mental health and wellbeing early in pregnancy and following the birth to help detect potential problems and prevent serious adverse outcome. However, identification of mental health problems in pregnancy may be less than 50 %.

The aim of the study was to find out which women are asked about their mood and mental health during pregnancy and postnatally, and about offer and uptake of treatment.

**Methods:**

Secondary analysis of a national maternity survey carried out in 2014 which asked about sociodemographic factors, care in pregnancy, childbirth, and the postnatal period with specific questions on emotional and mental health.

**Results:**

The usable response rate to the survey was 47 % (4571 women). Most women recalled being asked about their mental health in pregnancy (82 %) and in the postnatal period (90 %). However, antenatally, Asian and older women were less likely to be asked and to be offered treatment. In the postnatal period, differences were more marked. Non-white women, those living in more deprived areas, and those who had received less education were less likely to be asked about their mental health, to be offered treatment, and to receive support. Women with a trusting relationship with their midwife were more likely to be asked about their mental health.

**Conclusion:**

The inequities described in this study suggest that the inverse care law is operating in relation to this aspect of maternity care. Those women most likely to be in need of support and treatment are least likely to be offered it and may be at risk of serious adverse outcomes.

**Electronic supplementary material:**

The online version of this article (doi:10.1186/s12888-016-1029-9) contains supplementary material, which is available to authorized users.

## Background

Pregnancy and the early postnatal period are critical and often stressful times in the lives of women and their families [1]. At such a time of potential vulnerability a degree of worry, anxiety and low mood is normal [2], particularly in primiparous women, especially if the pregnancy is unplanned. It is also a time when women are likely to be in more frequent contact with healthcare professionals than usual. There is thus increased opportunity for identification, diagnosis and treatment of problems. However, women with mental health problems may be less willing to access care and anxious about disclosing their situation or history due to fear of stigma, labelling, and losing custody of the child [3]. Women with existing mental health conditions may also be socially isolated [4].

Mental health problems during pregnancy and following birth include a wide range of disorders which vary in severity [1]. Depression and anxiety, which may occur both antenatally and postnatally, occur in about 15 % of women [5, 6], and are frequently co-morbid [2]. Women with a previously existing mental health problem may require different medication during pregnancy or when breastfeeding, and for some conditions (e.g., bipolar disorder) there is an increased risk of an episode in the early postnatal period. Generalised anxiety disorder and adjustment disorder tend to be more severe during pregnancy and the early postnatal period [1, 7].

There is an increased risk of adverse outcome for both the mother and baby associated with mental health problems. For example, rates of prematurity and low birth weight are increased in babies of depressed women, especially if untreated [8]. Children of depressed mothers are also at increased risk of attachment difficulties, poor mother-infant relationships and developmental difficulties [9]. At the extreme, rates of suicide are higher in women with mental health problems [10] and mental health problems contributed to almost a quarter of maternal deaths in England between 2011 to 2013 [11]. It is thus essential that emotional and mental health issues are discussed with all women both in pregnancy and in the postnatal period.

The association between mental health and adverse outcome is moderated by socioeconomic factors, adversity, education, smoking and domestic abuse [12, 13]. Some of these factors may be amenable to intervention. Psychological and pharmacological treatments have been demonstrated to be effective [1] although they are not always acceptable.

Despite frequent and universal contact during pregnancy and in the early postnatal period, identification of mental health problems is thought to be as low as 50 % [1, 14, 15]. In 2014, the National Institute for Health and Care Excellence (NICE) recommended that a general discussion regarding mental health and wellbeing take place with all women both at the first contact in pregnancy and in the early postnatal period, and that questions about emotional and mental health are asked at each contact. NICE recommended that health professionals should consider asking the ‘Whooley’ questions [1]. These are: *‘During the last month, have you often been bothered by feeling down, depressed or hopeless?’* and *‘During the last month have you often been bothered by having little interest or pleasure in doing things?’* [16] If either of the Whooley questions elicits a positive response, it can be followed up with the Arroll question: *‘Is this something with which you would like help?’* [17] This brief screening has been criticised for its low sensitivity and specificity [18] although in that particular study the questions were asked in a self-completion format rather than being asked by a health professional. However, the Whooley-Arroll screening has the benefit of brevity, no additional resources are required, and it can be used both antenatally and postnatally.

It is not known how widely these or other screening tests are used or even what proportion of women have discussions with health professionals about their emotional and mental health. The aim of this study was to find out whether women are asked about their mood and mental health during pregnancy and in the postnatal period. Specifically, the research questions were:

- Which women are asked about their emotional and mental health at these times?
- Who is offered treatment?
- Who takes up the offer and receives support, advice, and/or treatment?

## Methods

This study involved secondary analysis of a national maternity survey carried out in 2014 [19]. Women who gave birth in the first half of January 2014 were randomly selected from birth registration statistics by staff at the Office for National Statistics (ONS). Women were excluded if they were aged less than 16 years or their baby had died. The questionnaire, together with a letter, information leaflet and sheet in 18 non-English languages encouraged women to complete the questionnaire (by phone with the help of an interpreter if necessary) and return it in the Freepost envelope. The questionnaire could also be completed online. Using a tailored reminder system [20] up to three reminders were sent as required.

Women were asked about events, care and experience of pregnancy, labour and birth and about the postnatal period, and questions about sociodemographic characteristics. They were asked if, at the time of pregnancy booking or a few weeks later, they had been asked about their current and past (including family history) emotional and mental health, and who asked them. If they had a mental health problem during pregnancy, they were asked whether they were *offered* treatment, and whether they *received* support, advice and/or treatment. Similarly, in the postnatal section, women were asked whether they had experienced a mental health problem since the birth and whether they had received support, advice and/or treatment.

ONS provided information about each woman’s age group, country of birth, marital status, and an area based measure, the Index of Multiple Deprivation (IMD) in quintiles, and whether or not she had responded to the questionnaire, which enabled comparison of responders and non-responders.

A descriptive analysis was carried out using raw percentages to establish how guidelines were being followed and to support service planning. As there was likely to be overlap between different sociodemographic factors, binary logistic regression was used to estimate the extent of this and to determine the main drivers for any differences seen. The results of the logistic regressions are shown in the Additional file 1. Ethical approval for the survey was obtained from the NRES committee for Yorkshire and The Humber – Humber Bridge (REC reference 14/YH/0065).

## Results

Completed returns were received from 4571 women representing a 47 % usable response rate. Compared to non-respondents, women who completed the questionnaire were significantly more likely to be older, married, living in a less deprived area and born in the UK. Three percent of respondents were sole registrants of the birth compared to 8 % of non-respondents, 24 % were born outside the UK compared to 30 % of non-respondents, and 20 % were resident in the most deprived quintile compared to 34 % of non-respondents [19].

### Questions about emotional and mental health in pregnancy

A total of 4521 women answered the questions relating to mental health in pregnancy. Of these women 82 % recalled having being asked about their current, and 84 % about their past emotional and mental health and family history. The questions were generally asked by a midwife (73 %), sometimes by the GP (10 %). There was little significant variation by sociodemographic factors in univariate analysis (Table 1): women of different age, level of education and living in areas varying by deprivation were equally likely to have been asked about their mental health during the pregnancy. However, Asian women and women who were multiparous were significantly less likely to be asked about their antenatal emotional and mental health or their past mental health, and women aged 40 years or more were also significantly less likely to be asked about their past mental health and family history. In logistic regression, parity was no longer significant but differences by ethnicity and age remained. Women in more deprived quintiles were more likely to be asked about past mental health problems (Additional file 1: Table S1).

**Table 1:** Characteristics of women asked at antenatal booking about their mental health and offered treatment

	Current mental health		Past/family history		Offered treatment	
	No.	%	No.	%	No.	%
Age of mother (yrs)						
16–19 (101)	80	80.8	82	82.8	5	17.2
20–24 (538)	431	80.9	460	87.8	52	35.1
25–29 (1228)	1021	83.9	1036	86.2	113	38.6
30–34 (1587)	1286	82.1	1308	84.1	104	36.4
35–39 (874)	705	81.2	696	81.5	52	35.6
40+ (241)	180	75.9	176	73.9	11	25.6
Total (4569)	3703	81.9	3758	84.0**	337	35.7
Index of Multiple Deprivation (quintile)						
1 (least deprived) (899)	716	80.2	723	81.5	49	40.2
2 (865)	708	82.9	724	84.7	46	35.1
3 (935)	763	82.3	774	84.5	69	39.0
4 (977)	803	83.3	811	85.1	75	33.2
5 (most deprived) (894)	714	80.8	727	84.3	97	33.6
Total (4570)	3704	81.9	3759	84.0	336	35.6
Parity						
Primiparous (2207)	1819	83.1	1856	85.5	136	32.9
Multiparous (2223)	1774	80.7	1798	82.9	182	38.2
Total (4430)	3593	81.9*	3654	84.2*	318	35.7
Ethnicity						
White (3715)	3045	82.6	3098	84.9	277	41.2
Mixed (87)	73	83.9	72	83.7	9	40.9
Asian (444)	331	75.7	341	79.1	29	19.6
Black (159)	130	83.9	124	81.0	7	17.9
Other (23)	18	78.3	17	77.3	3	37.5
Total (4428)	3597	82.0*	3652	84.1*	325	36.6**
Age left full-time education						
< 17 years (757)	601	80.6	607	82.4	83	39.7
17–18 years (1211)	980	81.7	1007	84.9	101	39.9
19+ yrs (2513)	2057	82.5	2083	84.3	144	32.2
still in education (3)	3	100.0	3	100.0	1	50.0
Total (4484)	3641	82.0	3700	84.1	329	36.1
Single mother						
No (3987)	3242	82.1	3286	84.1	277	36.7
Yes (591)	463	80.9	474	83.7	60	31.4
Total (4578)	3705	81.9	3760	84.0	337	35.6

*p < 0.05 **p < 0.01

Of the 946 women (21 %) who disclosed antenatal mental health problems, 36 % reported being offered treatment (Table 1). In univariate analysis, Asian and Black women were substantially and significantly less likely to report being offered treatment, 20 and 18 % respectively compared to 41 % of White women. Other sociodemographic factors were not significantly associated with the offer of treatment. These findings were confirmed in logistic regression but teenagers and women aged 40 or more were significantly less likely to be offered treatment (Additional file 1: Table S1).

Overall, 68 % of women who self-identified with mental health problems antenatally reported receiving support, 71 % received advice, and 45 % received treatment for mental health problems in pregnancy (Table 2). There was no significant variation by sociodemographic factors in those receiving advice or treatment at this time. However, in univariate analysis, women receiving support were significantly more likely to be White, living in a less deprived area, and more likely to have left full-time education aged 19 years or more. Where details of treatment were given, more than half of the women described support which included extra visits, information about specialist services, and counselling; about a third of women where mental health problems were identified received medication; cognitive behavioural therapy (CBT) was mentioned by only nine women. In logistic regression, Asian women were significantly less likely to have received support or advice, and women who had left full-time education aged 17 years or less were significantly less likely to have received support (Additional file 1: Table S2).

**Table 2:** Characteristics of women receiving support, advice and/or treatment for mental health problems in pregnancy

If mental health problem identified in pregnancy, woman received….						
	Support		Advice		Treatment	
	No.	%	No.	%	No.	%
Age of mother						
16–19 (10)	6	60.0	6	60.0	3	33.3
20–24 (53)	32	60.4	35	66.0	15	31.3
25–29 (107)	79	73.8	82	73.9	40	46.5
30–34 (83)	56	67.5	63	74.1	36	50.0
35–39 (46)	33	71.7	32	72.7	20	55.6
40+ (14)	8	57.1	11	61.1	5	41.7
Total (313)	214	68.4	229	71.3	119	45.2
Index of Multiple Deprivation (quintile)						
1 (least deprived) (36)	29	80.6	33	80.5	15	46.9
2 (45)	35	77.8	32	76.2	11	32.4
3 (64)	48	75.0	47	72.3	24	48.0
4 (77)	47	61.0	58	72.5	28	43.1
5 (most deprived) (90)	54	60.0	58	63.0	40	49.4
Total (312)	213	68.3*	228	71.3	118	45.0
Parity						
Primiparous (129)	84	65.1	91	67.9	44	40.7
Multiparous (170)	119	70.0	129	74.1	66	47.1
Total (299)	203	67.9	220	71.4	110	44.4
Ethnicity						
White (238)	177	74.4	188	76.4	92	46.2
Mixed (8)	4	50.0	4	57.1	3	60.0
Asian (36)	19	52.8	20	55.6	14	46.7
Black (10)	5	50.0	5	71.4	5	62.5
Other (2)	2	100.0	2	100.0	1	100.0
Total (294)	207	70.4*	219	73.5	115	47.3
Age left fullt-time education						
< 17 years (78)	45	57.7	51	64.6	31	44.9
17–18 years (82)	58	70.7	66	76.7	28	40.0
19+ yrs (145)	108	74.5	108	73.0	56	48.3
Total (305)	211	69.2*	225	71.9	115	45.1
Single mother						
No (233)	166	71.2	179	73.4	93	47.0
Yes (80)	48	60.0	50	64.9	26	40.0
Total (313)	214	68.4	229	71.3	119	45.2

* *p* < 0.05

### Questions about emotional and mental health in the postnatal period

Of the 4502 women who answered the questions about postnatal mental health, 90 % reported being asked about their emotional and mental health. In contrast to the antenatal question, there was significant and substantial variation across all sociodemographic characteristics (Table 3). Postnatally, women were significantly more likely to be asked about their emotional and mental health if they were older, living in a less deprived area, primiparous, White, had left full-time education aged 19 years or more and were not single mothers. This was confirmed in logistic regression except that age on leaving full-time education was no longer significant (Additional file 1: Table S3).

**Table 3:** Characteristics of women asked about their mental health, and receiving help in the postnatal period

	Asked about mental health		Support		Advice		Treatment	
	No.	%	No.	%	No.	%	No.	%
Age of mother (yrs)								
16–19 (99)	79	79.8	4	44.4	4	6	66.7	44.4
20–24 (527)	458	86.9	34	54.0	27	38	56.7	48.2
25–29 (1211)	1084	89.5	83	66.4	56	81	63.8	52.3
30–34 (1562)	1427	91.4	86	66.7	50	87	67.4	52.6
35–39 (862)	782	90.7	41	64.1	24	42	65.6	50.0
40+ (239)	213	89.1	15	60.0	4	12	54.5	23.5
Total (4500)	4043	89.8**	263	63.4	165	266	63.6	49.7
Index of Multiple Deprivation (quintile)								
1 (least deprived) (893)	825	92.4	50	70.4	28	48	69.6	52.8
2 (857)	787	91.8	40	66.7	19	37	61.7	45.2
3 (920)	844	91.7	60	69.0	38	62	72.1	60.3
4 (963)	859	89.2	57	60.0	36	58	61.1	45.6
5 (most deprived) (868)	729	84.0	56	54.4	44	61	56.0	45.8
Total (4501)	4044	89.8**	263	63.2	165	266	63.5	49.5
Parity								
Primiparous (2194)	2000	91.2	110	61.1	60	112	61.2	43.8
Multiparous (2208)	1958	88.7	143	65.3	97	143	65.3	53.0
Total (4402)	3958	89.9**	253	63.4	157	255	63.4	49.1
Ethnicity								
White (3698)	3399	91.9	217	67.4	134	216	67.3	53.8
Mixed (87)	74	85.1	9	90.0	3	7	70.0	50.0
Asian (437)	350	80.1	24	42.9	18	31	50.8	33.3
Black (155)	124	80.0	6	46.2	5	7	58.3	50.0
Other (23)	16	69.6	2	50.0	2	1	33.3	50.0
Total (4400)	3963	90.1**	258	63.7*	162	262	64.4	50.2
Age left full-time education								
< 17 years (752)	662	88.0	61	68.5	48	64	71.9	64.0
17–18 years (1205)	1058	87.8	67	60.9	44	67	62.0	52.4
19+ yrs (2495)	2286	91.6	132	62.6	70	133	61.6	41.7
Total (4455)	4009	90.0**	260	63.4	162	264	63.9	49.5*
Single mother								
No (3959)	3589	90.7	210	64	132	213	64	50
Yes (543)	456	84.0	53	60.2	33	53	61.6	47.8
Total (4502)	4045	89.8**	263	63.2	165	266	63.5	49.5

* *p* < 0.05 **p < 0.01

Women were also asked if they had talked to a health professional about what happened during labour and birth. Of the 4449 women who answered this question, 48 % had spoken with a health professional, most commonly to their health visitor (in 69 % of cases). Again, these women were significantly more likely to be more educated, living in a less deprived area and primiparous. They were also significantly more likely to have had a complicated delivery involving a caesarean section due to unforeseen problems. Women who had *not* talked to a health professional about labour and birth were asked if they would have liked to have done so. Of the 1439 women in this situation, 36 % indicated that they would have liked to have done so and, again, they were disproportionately primiparous, more educated, they were more likely to have had a complicated pregnancy and more likely to have experienced a caesarean section due to unforeseen problems. This was confirmed in logistic regression. Asian, but not Black, women were significantly less likely to have received support, advice or treatment. In addition, women aged 40 years or more were significantly less likely to receive treatment; conversely, women who had left full-time education aged less than 17 years were more likely to have received treatment (Additional file 1: Table S3).

As with the antenatal questions about mental health, women were asked if postnatally they had received support, advice and/or treatment for a mental health problem. Of those responding, about two-thirds of women received support (64 %) and advice (64 %) and half received treatment (50 %) (Table 3). The only significant variation by sociodemographic factors was that Asian and Black women were less likely to receive support postnatally, also that women who left full-time education aged less than 17 years were *more* likely to receive treatment. Where details of treatment were given, for more than half the women it consisted of medication and only seven women mentioned CBT. Support included extra health visitor and GP visits and postnatal support groups.

### Association with care factors

Unsurprisingly, women who booked early and saw the same midwife throughout their pregnancy were significantly more likely to feel able to talk to her about sensitive issues, and more likely to have been asked about their emotional and mental health. Women who were admitted to hospital during pregnancy, and presumably had more fragmented care, were less likely to be asked about their mental health although this finding was not statistically significant (Table 4).

**Table 4:** Association between care factors, satisfaction and questioning about emotional and mental health

In pregnancy…	Able to talk to HCP about sensitive issues			Asked about emotional and mental health	
	Always	Sometimes	Not at all	Yes	No
Booked at 12 weeks or less (3952)	47.1	35.2	17.7	82.4	17.6
Booked at >12 weeks (412)	41.4	36.2	22.4*	78.8	21.2
Same MW seen throughout (1575)	58.0	27.0	15.0	84.1	15.9
Not same MW seen (2888)	40.0	40.0	20.0**	80.9	19.1**
Hospital admission (809)	44.4	34.7	20.8	79.9	20.1
No hospital admission (3769)	46.9	35.5	17.6	82.4	17.6
In postnatal period after hospital discharge…		Overall satisfied		Asked about emotional and mental health	
		Yes	No	Yes	No
Had met all or some of the MWs before (2638)		80.4	19.6	91.2	8.8
Had not met any MWs before (1791)		73.0	27.0**	88.1	11.9**
Saw MWs as much as wanted (3381)		83.1	16.9	91.9	8.1
Would have liked to see them more (1037)		58.0	42.0**	84.6	15.4**
Received enough help with baby’s crying (1580)		86.3	13.7	92.4	7.6
Did not receive enough help with baby’s crying (1262)		61.9	38.1**	84.6	15.4**
Always had confidence and trust in MWs (3054)		86.6	13.4	91.8	8.2
Didn’t always have confidence and trust (1390)		57.2	48.8**	85.9	14.1**
Asked about emotional and mental health in pregnancy (3708)		-	-	92.4	7.6
Not asked about emotional and mental health in pregnancy (818)		-	-	78.4	21.6**

* *p* < 0.05 ** *p* < 0.01

MW midwife

Similarly in the postnatal period following hospital discharge, women who reported seeing a midwife they had met before, seeing a midwife as much as they wanted, receiving enough help with the baby and generally having confidence and trust in the midwives were also significantly more likely to have been asked about their emotional and mental health and to report being satisfied with their postnatal care. Women who were asked about their emotional and mental health in pregnancy were significantly more likely to be asked about it postnatally.

## Discussion

Childbirth is a major life event and women are potentially more vulnerable to mental health problems, particularly during the postnatal period. During pregnancy and postnatally there are opportunities to ask about mental health, to check and intervene if appropriate. While the results of this study suggest that more than four in five women were asked about their emotional and mental health – 82 % in pregnancy and 90 % in the postnatal period, the converse indicates that around one in five women were not asked about their emotional health antenatally and 1 in 10 postnatally. The concern about maternal mental health is reflected in the NICE guidelines and in the annual report of the Chief Medical Officer [21] which focused on the health of women and in which a responsibility for health professionals to ask all women about their mental health is emphasised.

During their pregnancy, a similar proportion of women, 84 %, were also asked about their past emotional and mental health and whether there was a family history of mental health problems. Women who described themselves as Asian were significantly less likely to be asked about their current or past mental health during pregnancy, and much less likely to be offered treatment. This is consistent with the results of a recent secondary analysis of the Born in Bradford data [22] which found that minority ethnic women were half as likely to have screening, and twice as likely to have a mental health problem missed as White British women.

In the postnatal period there was more marked variation. The findings clearly indicate that following birth White women, those living in less deprived areas and those who had received more education were more likely to be asked about their mental health, more likely to be offered treatment, and more likely to receive support than other women.

This is unfortunate as mental health problems tend to be most prevalent in disadvantaged parts of society [1]. It is also consistent with the Inverse Care law, that the availability of care tends to vary inversely with the needs of the population served [23]. This has been reported in many areas of health care including coronary surgery [24], management of depression [25], and overall service provision [26]. In the context of maternity services, the Inverse Care law has also been shown to operate in high income countries, for example, Canada where rural areas were disadvantaged [7], in the UK regarding choice of caesarean delivery without clinical indication [27], and in Australia regarding satisfaction and choice of antenatal care provider [28].

The disadvantage and, sometimes, discrimination faced by ethnic minority women has been highlighted in previous maternity care research [29–32] and may relate to unconscious bias and a lack of cultural awareness, but also stereotyping, and the practical difficulties of communicating with women who do not speak English. Muslim women may feel particularly inhibited from discussing such issues with male healthcare professionals [33].

Specifically regarding questioning women about their mental health, NICE has highlighted that there is a lack of information available to women prior to being asked about their mental health, regarding the consequences of particular responses, which may affect the honesty of answers [1]. There is also an unmet need for culturally appropriate information and support especially following diagnosis of a mental health problem, for the partner as well as for the women, and a lack of awareness of the different treatment options available [1]. This is reinforced by a qualitative study of women’s views of screening which reported that, although they were positive about being asked in general, they did not know what help might be available [34].

Limitations of this work include the 47 % response rate to the survey with under-representation of young and single women, those born outside the UK, and those living in areas of deprivation [19]. This is likely to have resulted in under-estimation of the proportions of women from disadvantaged groups not being asked about their mental health in the perinatal period. However, the questionnaires were well completed with missing values generally less than 3 %. The data relating to discussions of mental health are based on self-report, and may not agree with staff records. However, other research [35–37] indicates that salient events in childbearing are well-remembered. This study is strengthened by being population-based and by the large number of women who did respond.

Barriers to midwives in asking questions about mental health include the many tasks that have to be completed during a booking appointment, the lack of specific training, a lack of knowledge about referral for women needing additional help, and fragmented care [34].

This policy relevant study has demonstrated a lack of equity in assessment of and access to mental health support. Health professionals should endeavour to discuss emotional and mental health issues with *all* women both in pregnancy and the postnatal period. This could be facilitated by better training and more continuity of *care* and continuity of *carer*. In the NICE recommendations little distinction is made between these two concepts. Both are clearly important, for example, it is essential that specialist perinatal mental health services are integrated with the community to ensure continuity of care, but equally important to the women was having a known health professional to facilitate access, identification and treatment [1, 21, 38].

## Conclusions

Pregnancy and postnatal care represent a significant window of opportunity for identification, reassurance and intervention, with long-term implications for the safety and psychological wellbeing of women and their families as well as the costs associated with future care. Identification of women needing support and treatment is vital. This was recognised in the recent Maternity Review [38] in which this key role of maternity services was emphasised in improving access to mental health support for women, with review at every contact considered integral to personalised care.

## Additional file

Additional file 1: Table S1. Binary logistic regression of sociodemographic variables on whether women were asked about current, past or family history of mental health problems, and whether they were offered treatment during pregnancy. **Table S2.** Binary logistic regression of sociodemographic variables on whether women received support, advice or treatment during pregnancy. **Table S3.** Binary logistic regression of sociodemographic variables on whether women were asked about their mental health, and whether they received support, advice or treatment in the postnatal period. (DOCX 22 kb)
