# Supplementary material for: Who is actually asked about their mental health in pregnancy and the postnatal period? Findings from a national survey
Source: BMC Psychiatry. 2016 Sep 15;16:322. doi: 10.1186/s12888-016-1029-9 (PMC5025550; doi:10.1186/s12888-016-1029-9)
Supplement: Additional file 1: Table S1. — Binary logistic regression of sociodemographic variables on whether women were asked about current, past or family history of mental health problems, and whether they were offered treatment during pregnancy. Table S2. Binary logistic regression of sociodemographic variables on whether women received support, advice or treatment during pregnancy. Table S3. Binary logistic regression of sociodemographic variables on whether women were asked about their mental health, and whether they received support, advice or treatment in the postnatal period. (DOCX 22 kb) [file 12888_2016_1029_MOESM1_ESM.docx]

Additional data – Table 1 Binary logistic regression of sociodemographic variables on whether women were asked about current, past or family history of mental health problems, and whether they were offered treatment during pregnancy

|  | **Current MH problem** | | | **Past/family history** | | **Offered treatment** | |
| --- | --- | --- | --- | --- | --- | --- | --- |
|  | Odds Ratio | | 95% CI | Odds Ratio | 95% CI | Odds Ratio | 95% CI |
| Maternal age (years) | | |  |  |  |  |  |
| 16-19 | | 0.89 | (0.50, 1.55) | 0.80 | (0.45, 1.43) | 0.27 | (0.10, 0.78)* |
| 20-24 | | 0.88 | 0.67, 1.15) | 1.28 | (0.93, 1.75) | 0.77 | (0.49, 1.21) |
| 25-29 | | 1.12 | (0.91, 1.38) | 1.13 | (0.90, 1.42) | 0.78 | (0.54, 1.13) |
| 30-34 | | 1.00 |  | 1.00 |  | 1.00 |  |
| 35-39 | | 0.95 | (0.76, 1.19) | 0.79 | (0.63, 0.99)* | 0.81 | (0.51, 1.27) |
| 40 or more | | 0.72 | (0.52, 1.02) | 0.53 | (0.38, 0.74)* | 0.34 | (0.14, 0.83)* |
| Index of multiple deprivation | | | |  |  |  |  |
| 1 | | 1.00 |  | 1.00 |  | 1.00 |  |
| 2 | | 1.25 | (0.97, 1.61) | 1.23 | (0.95, 1.60) | 0.80 | (0.46, 1.38) |
| 3 | | 1.17 | (0.92, 1.49) | 1.24 | (0.96, 1.59) | 1.14 | (0.69, 1.91) |
| 4 | | 1.29 | (1.00, 1.65) | 1.43 | (1.10, 1.86) | 0.87 | (0.53, 1.44) |
| 5 (most deprived) | | 1.10 | (0.85, 1.43) | 1.32 | (1.00, 1.74)* | 0.95 | (0.58, 1.56) |
| Parity | |  |  |  |  |  |  |
| Primiparous | | 1.00 |  | 1.00 |  | 1.00 |  |
| Multiparous | | 0.88 | (0.75, 1.04) | 0.91 | (0.76, 1.08) | 1.23 | (0.90, 1.67) |
| Ethnicity | |  |  |  |  |  |  |
| White | | 1.00 |  | 1.00 |  | 1.00 |  |
| Mixed | | 1.08 | (0.60, 1.94) | 0.88 | (0.49, 1.58) | 0.84 | (0.34, 2.08) |
| Asian | | 0.67 | (0.52, 0.86)* | 0.67 | (0.51, 0.88)* | 0.29 | (0.18, 0.47)* |
| Black | | 1.09 | (0.68, 1.75) | 0.76 | (0.48, 1.20) | 0.23 | (0.08, 0.62) |
| Age left full-time education | | | |  |  |  |  |
| <17 years | | 0.92 | (0.74, 1.14) | 0.80 | (0.64, 1.01) | 1.15 | (0.80, 1.63) |
| 17 or more | | 1.00 |  | 1.00 |  | 1.00 |  |
| Single mother | | |  |  |  |  |  |
| Yes | | 0.96 | (0.74, 1.24) | 0.93 | (0.71, 1.22) | 1.08 | (0.71, 1.63) |
| No | | 1.00 |  | 1.00 |  | 1.00 |  |

** p<0.05*

Additional data – Table 2 Binary logistic regression of sociodemographic variables on whether women received support, advice or treatment during pregnancy

|  | | **Received support** | | | **Received advice** | | | **Received treatment** | | |
| --- | --- | --- | --- | --- | --- | --- | --- | --- | --- | --- |
|  | | Odds  Ratio | | 95% CI | Odds Ratio | | 95% CI | Odds Ratio | | 95% CI |
| Maternal age (years) | | |  | |  |  | |  |  | |
| 16-19 | 0.70 | | (0.15, 3.31) | | 0.72 | (0.15, 3.41) | | 0.60 | (0.12, 3.00) | |
| 20-24 | 0.57 | | (0.25, 1.29) | | 0.80 | (0.35, 1.84) | | 0.44 | (0.19, 1.02) | |
| 25-29 | 0.66 | | (0.32, 1.36) | | 0.91 | (0.44, 1.90) | | 1.01 | (0.50, 2.04) | |
| 30-34 | 1.00 | |  | | 1.00 |  | | 1.00 |  | |
| 35-39 | 0.68 | | (0.28, 1.64) | | 0.61 | (0.25, 1.46) | | 1.85 | (0.77, 4.46) | |
| 40 or more | 0.29 | | (0.08, 1.09) | | 0.31 | (0.09, 1.03) | | 0.77 | (0.19, 3.08) | |
| Index of multiple deprivation | | | | |  |  | |  |  | |
| 1 | 1.00 | |  | | 1.00 |  | | 1.00 |  | |
| 2 | 0.99 | | (0.31, 3.12) | | 0.75 | (0.24, 2.30) | | 0.48 | (0.16, 1.40) | |
| 3 | 1.13 | | (0.38, 3.34) | | 0.84 | (0.30, 2.37) | | 1.56 | (0.59, 4.10) | |
| 4 | 0.57 | | (0.20, 1.57) | | 0.85 | (0.31, 2.39) | | 0.99 | (0.39, 2.51) | |
| 5 (most deprived) | 0.54 | | (0.19, 1.51) | | 0.48 | (0.17, 1.32) | | 1.75 | (0.67, 4.62) | |
| Parity |  | |  | |  |  | |  |  | |
| Primiparous | 1.00 | |  | | 1.00 |  | | 1.00 |  | |
| Multiparous | 1.39 | | (0.75, 2.59) | | 1.44 | (0.79, 2.64) | | 0.86 | (0.46, 1.58) | |
| Ethnicity |  | |  | |  |  | |  |  | |
| White | 1.00 | |  | | 1.00 |  | | 1.00 |  | |
| Mixed | 0.37 | | (0.08, 1.64) | | 0.39 | (0.08, 1.92) | | 1.68 | (0.26, 10.84) | |
| Asian | 0.35 | | (0.15, 0.78)* | | 0.38 | (0.17, 0.85)* | | 0.53 | (0.21, 1.34) | |
| Black | 0.21 | | (0.04, 1.10) | | 0.49 | (0.08, 3.23) | | 1.23 | (0.21, 7.32) | |
| Age left full-time education | | | | |  |  | |  |  | |
| <17 years | 0.52 | | (0.28, 0.97) | | 0.67 | (0.35, 1.26) | | 0.95 | (0.49, 1.83) | |
| 17 or more | 1.00 | |  | | 1.00 |  | | 1.00 |  | |
| Single mother | | |  | |  |  | |  |  | |
| Yes | 0.99 | | (0.51, 1.94) | | 0.94 | (0.47, 1.87) | | 0.70 | (0.34, 1.42) | |
| No | 1.00 | |  | | 1.00 |  | | 1.00 |  | |

** p<0.05*

Additional data – Table 3 Binary logistic regression of sociodemographic variables on whether women were asked about their mental health, and whether they received support, advice or treatment in the postnatal period

|  | **Asked about mental health** | | **Received support** | | **Received advice** | | **Received treatment** | |
| --- | --- | --- | --- | --- | --- | --- | --- | --- |
|  | Odds Ratio | 95% CI | Odds Ratio | 95% CI | Odds Ratio | 95% CI | Odds Ratio | 95% CI |
| Maternal age (years) | |  |  |  |  |  |  |  |
| 16-19 | 0.32 | (0.18, 0.57)* | 0.32 | (0.08, 1.37) | 0.84 | (0.18, 3.85) | 0.55 | (0.12, 2.44) |
| 20-24 | 0.59 | (0.42, 0.83)* | 0.56 | (0.28, 1.13) | 0.75 | (0.38, 1.47) | 0.96 | (0.45, 2.04) |
| 25-29 | 0.82 | (0.62, 1.07) | 0.93 | (0.52, 1.67) | 1.26 | (0.71, 2.23) | 1.00 | (0.54, 1.84) |
| 30-34 | 1.00 |  | 1.00 |  | 1.00 |  | 1.00 |  |
| 35-39 | 0.94 | (0.69, 1.28) | 0.71 | (0.36, 1.39) | 0.92 | (0.47, 1.80) | 0.77 | (0.37, 1.61) |
| 40 or more | 0.86 | (0.54, 1.38) | 0.60 | (0.23, 1.59) | 0.51 | (0.19, 1.36) | 0.25 | (0.07, 0.87) |
| Index of multiple deprivation | | |  |  |  |  |  |  |
| 1 | 1.00 |  | 1.00 |  | 1.00 |  | 1.00 |  |
| 2 | 1.03 | (0.71, 1.48) | 0.74 | (0.34, 1.59) | 0.60 | (0.28, 1.30) | 0.67 | (0.28, 1.59) |
| 3 | 1.04 | (0.73, 1.48) | 1.03 | (0.50, 2.16) | 1.14 | (0.54, 2.42) | 1.25 | (0.55, 2.83) |
| 4 | 0.97 | (0.69, 1.37) | 0.76 | (0.37, 1.55) | 0.73 | (0.35, 1.51) | 0.66 | (0.30, 1.46) |
| 5 (most deprived) | 0.70 | (0.50, 0.99)* | 0.63 | (0.30, 1.32) | 0.59 | (0.28, 1.22) | 0.63 | (0.29, 1.41) |
| Parity |  |  |  |  |  |  |  |  |
| Primiparous | 1.00 |  | 1.00 |  | 1.00 |  | 1.00 |  |
| Multiparous | 0.73 | (0.59, 0.91)* | 1.21 | (0.75, 1.93) | 1.12 | (0.71, 1.77) | 1.28 | (0.76, 2.15) |
| Ethnicity |  |  |  |  |  |  |  |  |
| White | 1.00 |  | 1.00 |  | 1.00 |  | 1.00 |  |
| Mixed | 0.51 | (0.27, 0.94)* | 3.93 | (0.47, 33.10) | 1.10 | (0.26, 4.59) | 0.67 | (0.12, 3.57) |
| Asian | 0.37 | (0.28, 0.50)* | 0.37 | (0.19, 0.71)* | 0.50 | (0.27, 0.94)* | 0.39 | (0.18, 0.80)* |
| Black | 0.43 | (0.27, 0.69)* | 0.46 | (0.13, 1.71) | 0.70 | (0.18, 2.67) | 0.97 | (0.22, 4.22) |
| Other | 0.20 | (0.08, 0.50)* | 0.49 | (0.07, 3.72) | 0.22 | (0.02, 2.58) | 0.92 | (0.12, 7.18) |
| Age left full-time education | | |  |  |  |  |  |  |
| <17 years | 0.86 | (0.66, 1.12) | 1.38 | (0.78, 2.43) | 1.75 | (1.75, 3.12)* | 2.26 | (1.22, 4.18)* |
| 17 or more | 1.00 |  | 1.00 |  | 1.00 |  | 1.00 |  |
| Single mother | |  |  |  |  |  |  |  |
| Yes | 0.72 | (0.54, 0.96)* | 1.08 | (0.61, 1.88) | 1.24 | (0.70, 2.19) | 1.10 | (0.59, 2.14) |
| No | 1.00 |  | 1.00 |  | 1.00 |  | 1.00 |  |

** p<0.05*
